# Antithrombin III Alleviates Myocardial Ischemia/Reperfusion Injury by Inhibiting Excessive Autophagy in a Phosphoinositide 3-Kinase/Akt-Dependent Manner

**DOI:** 10.3389/fphar.2019.00516

**Published:** 2019-05-10

**Authors:** Kai-yu Huang, Jiao-ni Wang, Ying-ying Zhou, Shao-ze Wu, Lu-yuan Tao, Yang-pei Peng, Jia-qun Que, Yang-jing Xue, Kang-ting Ji

**Affiliations:** ^1^ Department of Cardiology, The Second Affiliated and Yuying Children’s Hospital of Wenzhou Medical University, Wenzhou, China; ^2^ Department of Endocrinology, The Second Affiliated and Yuying Children’s Hospital of Wenzhou Medical University, Wenzhou, China; ^3^ Department of Cardiology, Zhejiang Hospital, Hangzhou, China; ^4^ Department of Cardiology, Taizhou First People’s Hospital, Taizhou, China

**Keywords:** antithrombin III, myocardial, ischemia reperfusion, autophagy, apoptosis

## Abstract

Autophagy is fundamental to myocardial ischemia/reperfusion (I/R) injury. Antithrombin III (AT) has been shown to protect cardiomyocytes against I/R injury; however, it is unknown whether it modulates autophagy. The objective of this study was to investigate whether AT regulates autophagy during I/R injury and, if so, to identify the potential mechanism involved. Our study showed that AT attenuated I/R injury *in vivo* and hypoxia/reoxygenation (H/R) injury *in vitro*. Autophagy was increased both in H9C2 cardiomyocytes during H/R injury and in mouse hearts following I/R injury. The stimulation of autophagy by rapamycin attenuated the protective effect of AT against H9C2 cell injury, indicating that autophagy is involved in the protective role of AT. Furthermore, the cardioprotective effects of AT were abolished by A6730, a specific Akt inhibitor. This study shows that AT exhibits cardioprotective effects by modulating autophagy during I/R injury in a phosphoinositide 3-kinase/Akt-dependent manner.

## Introduction

Myocardial infarction (MI) caused by coronary artery occlusion has become a principal cause of morbidity and mortality worldwide (Fox et al., 2007). Primary angioplasty and coronary artery bypass surgery, as well as ingestion of anticoagulant and thrombolytic drugs, are the classical treatment strategies for the recovery of blood flow in response to myocardial ischemia (Ferdinandy et al., 2007). Despite their safety and efficacy, the major drawback of these treatments is the irreversible injury to the myocardium *via* a pathological phenomenon known as ischemia/reperfusion (I/R), which induces autophagy both in animal and cell models (Sadoshima, 2008; Zhao et al., 2013). Autophagy is believed to play a beneficial role in ischemic hearts, as it improves cell survival and cardiac function. However, excessive autophagy has been shown to aggravate cell death *via* the overexpression of beclin-1 during reperfusion (Nakai et al., 2007; Nishino et al., 2008). Several studies have shown that autophagy provides cardioprotective benefits (Hamacher-Brady et al., 2006; Kanamori et al., 2011). One previous study explored the potential mechanisms of antithrombin III (AT) that contribute to myocardial protection after I/R injury, including anti-inflammation, the modulation of substrate metabolism (Ma et al., 2015), and the upregulation of genes related to apoptosis and cell cycle arrest (Zhang et al., 2006). However, whether autophagy is a protective response of AT in the myocardium suffering from I/R injury remains unknown.

## Materials and Methods

**Figure 1:**
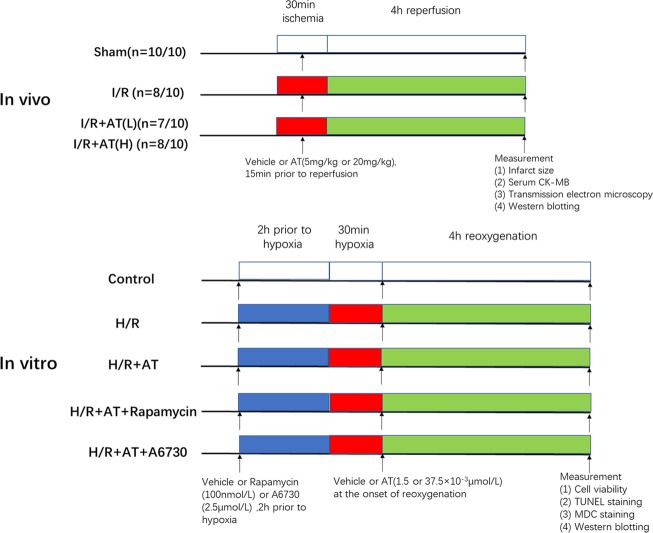
Experimental design. *In vivo*, I/R + AT(L) indicates the myocardial I/R injury group treated with a low dose of AT (5 mg/kg), and I/R + AT(H) indicates myocardial I/R injury group treated with a high dose of AT (20 mg/kg). (n = number 1/number 2; the former number indicates the number of mice in the group that underwent the surgery and survived, and the latter number indicates the total number of mice in the group). *In vitro*, H/R + AT(L) indicates the H9C2 cell H/R injury group exposed to a low dose of AT (1.5 × 10^–3^ μmol/L) and H/R + AT (H) indicates the H9C2 cell H/R injury group exposed to a high dose of AT (37.5 × 10^–3^ μmol/L), H/R + AT + rapamycin indicates the H9C2 cell group pretreated with rapamycin (100 nmol/L) for 2 h, followed by the induction of hypoxia and then treatment with a high dose of AT at the onset of reoxygenation, and H/R + AT + A6730 indicates the H9C2 cell group pretreated with A6730 (2.5 μmol/L) for 2 h, followed by the induction of hypoxia and then treatment with a high dose of AT at the onset of reoxygenation.

The experimental design of our study was presented in Figure 1.

### Reagents and Antibodies

Antithrombin, rapamycin, A6730, Evans blue, and triphenyltetrazolium chloride (TTC) were purchased from Sigma-Aldrich (St. Louis, MO). Anti-beclin-1 (1:1000), anti-GAPDH (1:5000), anti-protein kinase B (Akt; 1:1000), anti-phosphorylated (p)-Akt (Ser473,1:1000), anti-autophagy protein microtubule-associated protein 1 light chain-3B (LC3B; 1:1000), anti-B-cell lymphoma 2 (Bcl-2; 1:1000), anti-Bcl-2 associated x (Bax; 1:1000), and anti-p62 (1:1000) antibodies were obtained from either Cell Signaling Technology (Danvers, MA) or Abcam (Cambridge, UK). Phenylmethane sulphonyl fluoride (PMSF), the terminal deoxynucleotidyl transferase dUTP nick end labeling (TUNEL) assay kit, and the bicinchoninic acid protein quantification kit were obtained from Beyotime (Shanghai, China). The creatine kinase (CK) assay kit was purchased from the Jiancheng Bioengineering Institute (Nanjing, China). Goat anti-rabbit secondary antibodies were purchased from Biosharp (Guangzhou, China).

### Animals and H9C2 Myocyte Culture

A total of 80 male C57BL/6 mice (6–7 weeks old), weight 20–25 g, were obtained from the SLAC Laboratory Animal Centre of Shanghai (Shanghai, China). The Animal Care and Use Committee at the Wenzhou Medical College approved the study (NO: wydw2018-0056). The current study procedures and surgical operations were conducted in accordance with the Guidelines for the Care and Use of Laboratory Animals (National Institutes of Health, Bethesda, MD). H9C2 cells, obtained from the American Type Culture Collection (Manassas, VA), were cultured in Dulbecco’s modified Eagle’s medium (DMEM) containing 4.5 g/L glucose, 10% fetal bovine serum, and 1% penicillin/streptomycin at 37°C in a humidified atmosphere. Before different treatments to H9C2, the cell confluence should reach to 70–80%.

### Ischemia/Reperfusion Procedure

C57BL/6 mice were assigned randomly to one of four groups. In the sham group, identical operations without left anterior descending artery (LAD) occlusion were performed. Different concentrations of AT [4 or 20 mg/kg, dissolved in saline, corresponding to low (L) concentration and high (H) concentration, respectively] were administered *via* intravenous injection 15 min prior to reperfusion (Ma et al., 2015). Surgery was performed as described previously (Wu et al., 2017). Briefly, isoflurane was used to anesthetize the mice. Following lateral cutting, the heart was exposed under the third or fourth rib. A 7-0 silk suture was used to occlude the distal one-third of the entire LAD. Following 30 min of ischemia, the occlusion was released to allow reperfusion for 4 h.

### Detection of Infarct Size and Area at Risk in the Myocardium

TTC staining was used to detect the myocardial infarct size induced by I/R injury. Briefly, 2% Evans blue was injected into the postcava following completion of the I/R procedure and re-occlusion of the LAD. The animals were then sacrificed using an overdose of chloral hydrate. The LV tissue were removed immediately, cut into 2 mm slices, and placed in 1% TTC for 10 min at 37°C in the dark. The slices were incubated in 4% formalin for another 24 h. Normal areas of the myocardium were stained blue, the infarcted areas were pale gray or white, and the areas at risk were stained red. The images were analyzed using Image Pro software (Media Cybernetics, Inc., Bethesda, MD), and infarct size from five slices of each heart was calculated using a method as previously described (Xie et al., 2014).

### Measurement of Creatine Kinase-Muscle/Brain Fraction Release

After reperfusion, blood samples were obtained from the abdominal aorta and centrifuged at 3,000 *g* for 10 min to separate the serum. The level of CK-muscle brain (MB) was assayed in serum according to the manufacturer’s instructions using a microplate reader (Thermo Fisher Scientific, Shanghai, China).

### Hypoxia/Reoxygenation in H9C2 Cells

H9C2 cells are widely used to evaluate cardiomyocyte H/R injury (Wang et al., 2016b). H/R induction was performed as described previously (Yu et al., 2015). Briefly, H9C2 cells were exposed to an hypoxia buffer containing (in mmol/L) 137 NaCl, 12 KCl, 0.49 MgCl_2_, 0.9 CaCl_2_, 4 HEPES, 10 deoxyglucose, 0.75 sodium dithionate, and 20 lactate (pH 6.5) for 30 min in a humidified cell culture incubator (21% O_2_, 5% CO_2_, 37°C). Following hypoxia for 30 min, cells were returned to normal culture medium with or without various concentrations of AT for 4 h. Prior to H/R injury, cells were pretreated with 100 nmol/L rapamycin or 2.5 μmol/L A6730, an Akt1/2 inhibitor, for 2 h.

### Assessment of Cell Viability

The Cell Counting Kit-8 (CCK-8) assay (Solarbio, Beijing, China) was used to assess cell viability. H9C2 cells (5 × 10^3^ cells/well) were grown in 96-well plates and treated using the conditions as described above. Following treatment, the medium of each group was replaced with 90 μl serum-free DMEM plus 10 μl CCK-8 for 2 h, and then the absorbance was measured at 450 nm.

### TUNEL Staining

TUNEL staining is a flexible method used to measure apoptosis. Following H/R injury, cells were incubated in 4% formalin for 30 min and 0.3% Triton X-100 for 10 min. Cells were then stained with TUNEL working solution and 4′6-diamidino-2-phenylindole (DAPI) in the dark according to the manufacturer’s instructions. Samples were washed with phosphate-buffered saline (PBS) three times after each step. A fluorescence microscope (Olympus Inc., Tokyo, Japan) was used to assess changes in apoptosis.

### Transmission Electron Microscopy

Following induction of I/R, tissues (1 mm^3^) from fresh hearts were incubated in 2.5% glutaraldehyde for 4 h. Following fixation in 1% osmium tetroxide for 1 h, samples were dehydrated with an increasing concentration of alcohol. Finally, samples were embedded and stained. Transmission electron microscopy (FEI, Hillsboro, OR) was used to analyze the samples.

### Monodansylcadaverine Staining

Monodansylcadaverine (MDC; Sigma-Aldrich) is commonly used to stain autophagic vacuoles (Wang et al., 2016a). H9C2 cells were seeded in 24-well plates with sterile cover slips and incubated with 0.05 mM MDC for 1 h in the dark. Cells were then washed with PBS and scanned using a fluorescence microscope (Olympus).

### Western Blotting

Samples were digested with radioimmunoprecipitation assay buffer containing 1 mM PMSF. Western blot analysis was performed on the extracted proteins as described previously (Chen et al., 2016). Following incubation with primary antibodies, the membranes were washed with tris-buffered saline containing Tween-20 and incubated with the appropriate secondary antibodies. The loading control of this study is anti-GAPDH (1:5000). Each membrane was then visualized using the Electrochemiluminescence Plus reagent (Bio-Rad Laboratories, Hercules, CA) and quantified using Image Lab 3.0 software (Bio-Rad Laboratories).

### Statistical Analyses

Statistical analysis was performed by the SPSS software 21.0. Statistically significant differences were evaluated by Student *t*-test (for two groups) and one-way ANOVA with Tukey’s post hoc test or nonparametric Kruskal-Wallis test followed by the Bonferroni test (for multi-group). The data are expressed as mean ± SD. *p* < 0.05 was considered significant.

## Results

### Antithrombin III Treatment Attenuates Myocardial Injury Caused by Ischemia/Reperfusion *in vivo*

**Figure 2:**
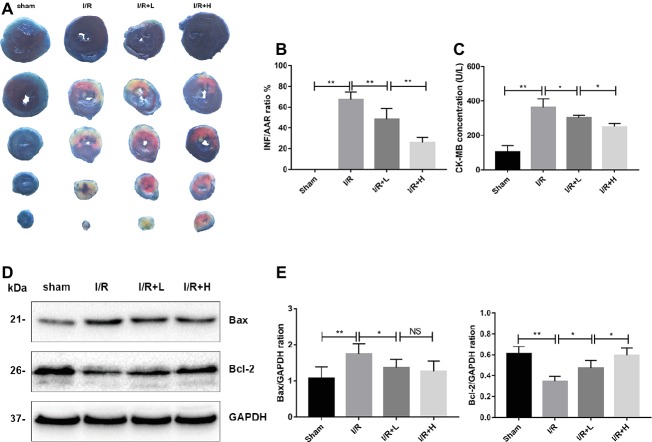
AT treatment attenuates mouse I/R injury in vivo. **(A,B)** Myocardial infarct size was identified by TTC staining. The infarct size increased in the I/R group, whereas AT treatment (4 or 20 mg/kg) reduced the infarct size in a dose-dependent manner compared with the I/R group. **(C)** CK-MB was significantly higher in the I/R group than that in the control group, and AT significantly reduced the release of CK-MB from the myocardium after I/R injury. **(D,E)** Immunoblot analysis for Bax and Bcl-2 extracted from mouse LV tissues. Significant differences between the treatment and control groups are indicated as **p < 0.01, *p < 0.05, *n* = 6. The values are expressed as the means ± SD (each experiment was repeated three independent times).

The myocardial infarct size was assessed to determine the protective role of AT following I/R injury. AT treatment diminished the myocardial infarct size in a dose-dependent manner (Figures 2A,B) (*p* < 0.01). Mice with I/R injury treated with AT showed a reduced CK-MB level in a dose-dependent manner (Figure 2C). To investigate whether the protective effect of AT was mediated by its anti-apoptotic role, the protein levels of Bax and Bcl-2 were assessed. As shown in Figures 2D,E, I/R injury significantly decreased the level of Bcl-2 (*p* < 0.01) but upregulated that of Bax (*p* < 0.05). These effects were reversed by AT treatment, indicating that AT can prevent apoptosis in the myocardium. Interestingly, there was no significant difference in the Bax level between low- and high-dose AT treatments. Further studies are needed to define the precise mechanism.

### Antithrombin III Treatment Reduces Autophagy Following Ischemia/Reperfusion Injury *in vivo*

**Figure 3:**
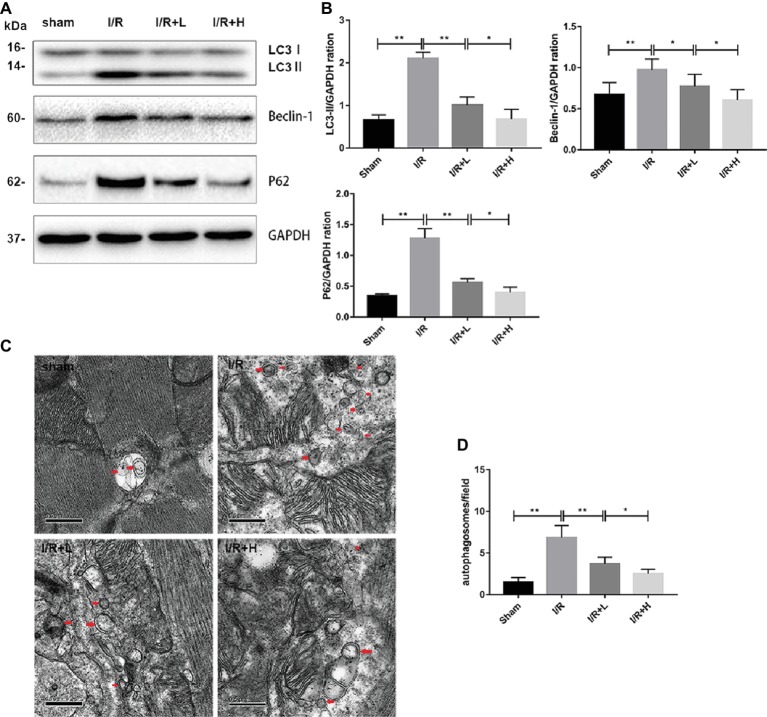
AT treatment reduces autophagy *in vivo*. **(A,B)** Western immunoblotting was performed using lysates from mouse LV tissues treated with AT (as described in Methods) to quantify the levels of LC3-II, beclin-1, and p62. **(C,D)** Transmission electron micrographs of LV tissue sections. Transmission electron micrographs (×25,000) of the myocardium showing autophagosomes surrounded by double membranes (arrows). Significant differences between the treatment and control groups are indicated as **p < 0.01, *p < 0.05, *n* = 6. The values are expressed as the means ± SD (each experiment was repeated three independent times).

Autophagy has recently emerged as a powerful platform for cell death after I/R injury. Following I/R injury, an upregulation of LC3-II and beclin-1 expression was observed in mouse LV tissues (Figures 3A,B) (*p* < 0.01), suggesting that autophagy might be activated by I/R injury. However, treatment with 4 or 20 mg/kg AT decreased the expression of LC3-II and beclin-1. The expression of p62, a marker of autophagic flux that is degraded in autophagosomes, was significantly increased in the myocardium during I/R injury, whereas AT attenuated the increased expression. The changes in the levels of LC3 and p62 indicate that I/R injury can increase the blocked autophagic flux *in vivo*, but AT can restore the autophagic flux and enhance the degradation of autophagosomes. Transmission electron microscopy was used to evaluate the morphological changes in autophagosomes. Following I/R injury, the number of autophagosomes was increased in the injured area compared with the uninjured area in the sham-treated control. AT treatment after I/R injury reduced the number of autophagosomes in a dose-dependent manner (Figures 3C,D) (*p* < 0.01), revealing that AT treatment can reduce the degree of autophagy induced by I/R injury.

### Antithrombin III Treatment Alleviates Hypoxia/Reoxygenation Injury in H9C2 Cells

**Figure 4:**
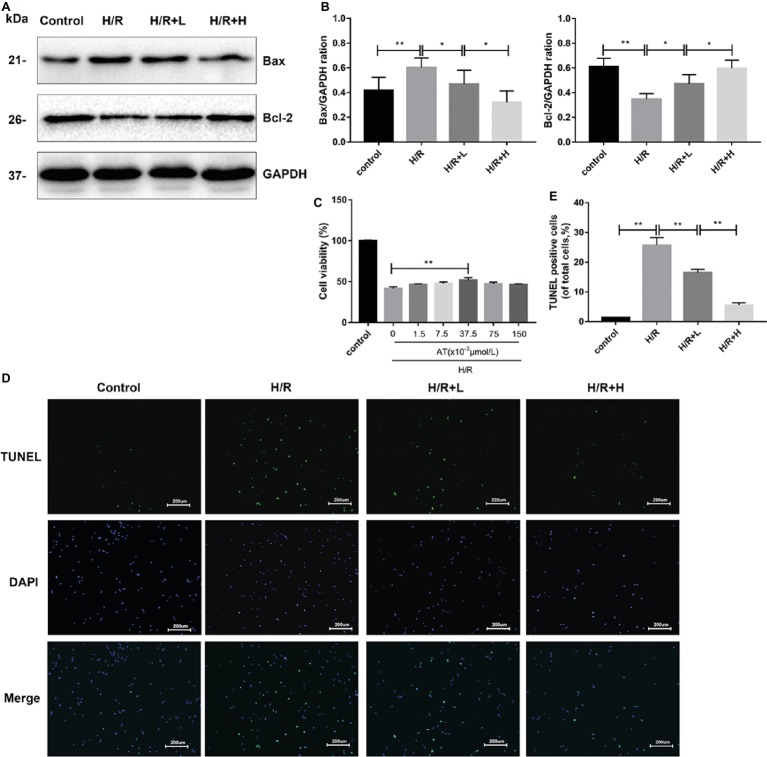
AT treatment alleviates H/R injury in H9C2 cells. **(A,B)** Western blotting was performed using lysates from H9C2 cells treated with AT to quantify the levels of apoptosis-related proteins. **(C)** Cell viability of H9C2 cells treated with different concentrations of AT during H/R injury. **(D,E)** TUNEL assay was performed using H9C2 cells as treated above (original magnification, ×100; scale bar, 200 μm). Significant differences between the treatment and control groups are indicated as **p < 0.01, *p < 0.05, *n* = 6. The values are expressed as the means ± SD (each experiment was repeated three independent times).

AT treatment enhanced the viability of H9C2 cells following H/R injury (Figure 4C). At a concentration of 37.5 × 10^–3^ μmol/L, AT significantly increased cell viability by about 24.9% (*p* < 0.01). AT was then used at low (L) and high (H) concentrations of 1.5 × 10^–3^ μmol/L and 37.5 × 10^–3^ μmol/L, respectively, in subsequent experiments. To assess cardiomyocyte injury following H/R, apoptosis was evaluated by Western blot analysis of apoptotic markers and TUNEL staining (Figures 4A,B,D,E). H/R injury markedly decreased the protein level of Bcl-2 (*p* < 0.01) but upregulated that of Bax (*p* < 0.01), which was reversed by AT treatment in a dose-dependent manner. TUNEL staining revealed that the proportion of apoptotic cells increased after H/R injury but decreased after AT treatment (Figures 4D,E) (*p* < 0.01). These results demonstrate that AT can alleviate cellular injury induced by H/R in a dose-dependent manner.

### Antithrombin III Treatment Inhibits Autophagy in H9C2 Cells During Hypoxia/Reoxygenation

**Figure 5:**
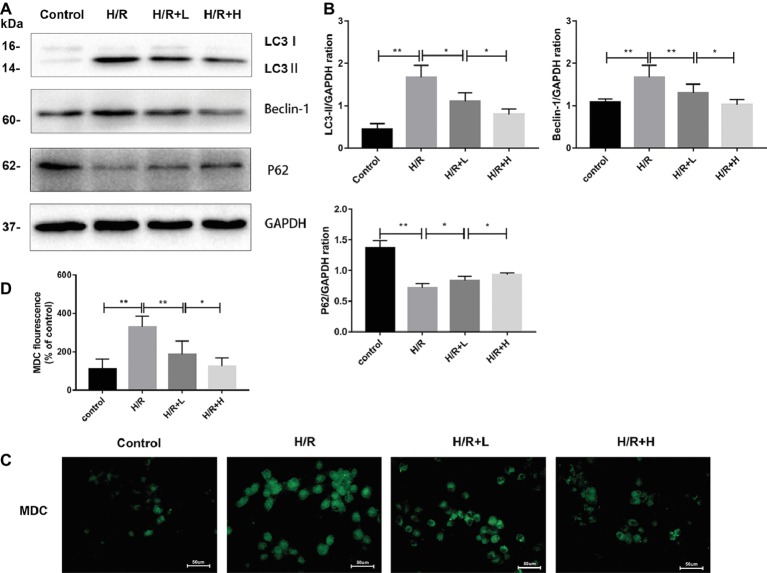
AT treatment inhibits autophagy in H9C2 cells following H/R injury. **(A,B)** Western immunoblotting was performed using lysates from H9C2 cells treated with AT to quantify the levels of LC3-II, beclin-1, and p62. **(C,D)** Autophagic vacuoles of H9C2 cells were stained with monodansylcadaverine (original magnification, ×400; scale bar, 50 μm). Significant differences between the treatment and control groups are indicated as **p < 0.01, *p < 0.05, *n* = 6. The values are expressed as the means ± SD (each experiment was repeated three independent times).

The H/R-induced upregulation of autophagy has been previously observed in H9C2 cells. In this study, the ability of AT to modulate H/R-mediated autophagy in H9C2 cells was assessed by MDC staining. The number of MDC-labeled vesicles was higher in the H/R group than that in the control group, whereas AT treatment decreased the number of MDC-labeled vesicles in a dose-dependent manner (Figures 5C,D) (*p* < 0.01). P62 attaches to mature autophagic vesicles and is degraded in autophagosomes. P62 expression was decreased in the H/R group, but AT treatment attenuated this effect in a dose-dependent manner. On the contrary, the expressions of LC3, a specific marker of the initiation of autophagy and the formation of autophagosomes, and beclin-1 were both increased in the H/R group. Similarly, these effects were reversed by AT in a dose-dependent manner (Figures 5A,B) (*p* < 0.01). Taken together, these results show that AT can inhibit autophagy induced by H/R injury in H9C2 cells.

### Antithrombin III-Mediated Cardiomyocyte Protection is Related to an Inhibition of Autophagy

**Figure 6:**
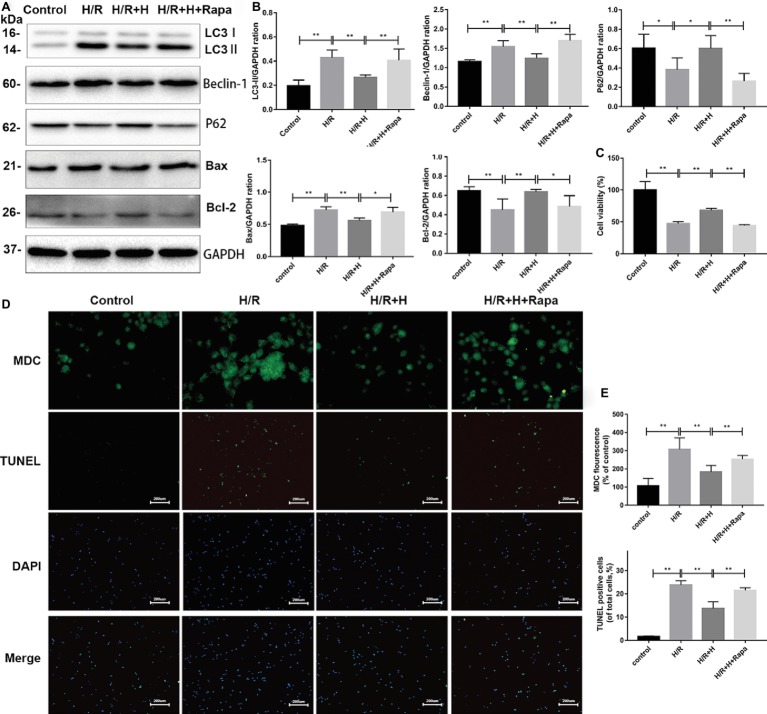
AT treatment alleviates H/R injury in H9C2 cells due to the attenuation of autophagy. **(A,B)** Representative western blots of autophagic marker proteins using H9C2 cell lysates. H9C2 cells were treated with 100 nmol/L rapamycin. **(C)** Cell viability of H9C2 cells. AT markedly increased cell viability in both the H/R group and rapamycin-treated group. **(D,E)** MDC staining (original magnification, ×400; scale bar, 50 μm) and TUNEL assay (original magnification, ×100; scale bar, 200 μm) were performed in H9C2 cells as treated above. Significant differences between the treatment and control groups are indicated as **p < 0.01, *p < 0.05, *n* = 6. The values are expressed as the means ± SD (each experiment was repeated three independent times).

Rapamycin (100 nmol/L), a specific agonist of autophagy, was used to verify the ability of AT to inhibit autophagy after H/R injury (Li et al., 2017). The CCK-8 assay was used to assess cell viability. The results indicated that the viability of cells was markedly decreased in both the H/R group and rapamycin-treated group. However, treatment with AT reversed these effects. As shown in Figure 6, the protein levels of LC3-II, beclin-1, and Bax were all decreased, whereas those of p62 and Bcl-2 were increased following AT treatment. Treatment with rapamycin prevented the increase of p62 and Bcl-2 expression. MDC and TUNEL staining revealed that autophagy and apoptosis were stimulated following H/R injury, and this phenomenon was reduced after treatment with AT. Cells pretreated with rapamycin exhibited a higher rate of apoptosis, revealing that the inhibition of autophagy prevented H/R-induced apoptosis as well (Figures 6D,E) (*p* < 0.01).

### AT Inhibits Hypoxia/Reoxygenation Injury in H9C2 Cells by Reducing Autophagy in a Phosphoinositide 3-Kinase/Akt-Dependent Manner

**Figure 7:**
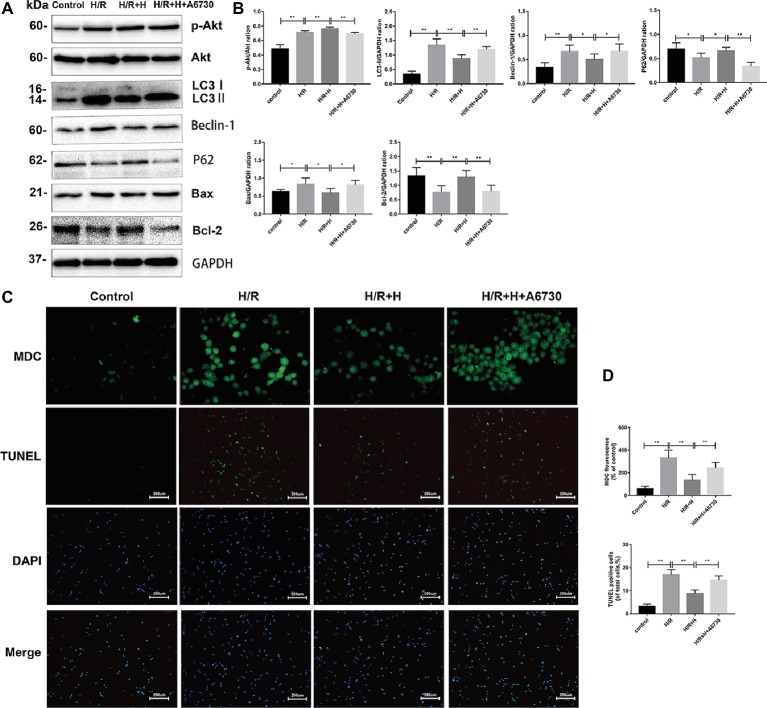
PI3K/Akt signaling is involved in the cardioprotective effects of AT. **(A,B)** Representative western blots of p-Akt, Akt, autophagic marker proteins, and apoptosis-related proteins using H9C2 cell lysates. **(C,D)** MDC staining (original magnification, ×400; scale bar, 50 μm) and TUNEL assay (original magnification, ×100; scale bar, 200 μm) were performed in H9C2 cells as treated above. Significant differences between the treatment and control groups are indicated as **p < 0.01, *p < 0.05, *n* = 6. The values are expressed as the means ± SD (each experiment was repeated three independent times).

Next, the potential signaling pathways that regulate autophagy and the protective effects of AT were explored. Compared with the control group, the H/R group showed markedly higher expression of p-Akt/Akt, indicating that H/R injury activates the phosphoinositide 3-kinase (PI3K)/Akt pathway. Interestingly, AT treatment increased the ratio of p-Akt/Akt. To confirm whether AT inhibits autophagy *via* activation of the PI3K/Akt signaling pathway, the effects of the specific Akt inhibitor A6730 were tested (Zheng et al., 2017). Treatment with A6730 at a concentration of 2.5 μmol/L decreased the p-Akt/Akt ratio and reversed the AT-mediated downregulation of beclin-1, LC3-II, and Bax expression as well as the upregulation of p62 and Bcl-2 expression following H/R injury (Figures 7A,B). MDC and TUNEL staining revealed that A6730 reversed the AT-mediated decreases in autophagy and apoptosis (Figures 7C,D).

## Discussion

In this study, we showed that (1) AT attenuated myocardial I/R injury in a dose-dependent manner, (2) I/R-induced autophagy was significantly suppressed by AT, which mediated the cardioprotective effects of AT, and (3) regulation of autophagy by the PI3K/Akt signaling pathway played an important role in the cardioprotective mechanism of AT. These results expand the evidence of the existing literature by illustrating the effects of AT on myocardial I/R injury and by providing further insights into the molecular mechanisms of AT protection from I/R injury in the myocardium. Several studies have shown that high levels of oxidative stress are produced in the myocardium and cardiomyocytes after I/R (Becker, 2004). It has also been reported that oxidative stress can induce autophagy and apoptosis (Hariharan et al., 2011; Ding et al., 2017). A previous study has shown that pathogenic oxidative stress triggered by I/R injury is attenuated by AT treatment. A strong relationship between AT and ULK1, a modulator of autophagy, was also reported previously (Ma et al., 2015). Consequently, we aimed to elucidate the association between AT and autophagy in this study.

Autophagy is the process of self-digestion that removes unnecessary proteins or organelles, and it sustains the normal morphology and function of cells, as in the case of myocardial ischemia (Gustafsson and Gottlieb, 2008). However, studies, such as the one conducted by Matsui et al. (2008), have shown that in the case of myocardial ischemic injury, autophagy induces cell survival, whereas reperfusion induces cell death. A large and growing body of literature has demonstrated that a moderate level of autophagy can provide a certain degree of protection in various pathologies, including myocardial I/R injury (Matsui et al., 2007), Alzheimer’s disease (Shin et al., 2014), and hepatitis (Vescovo et al., 2012). However, another study reported that the rapamycin-dependent activation of autophagy aggravated neonatal cardiomyocyte I/R injury (Xiao et al., 2015). The regulation of autophagy in cardiomyocytes can be an effective interventional target for I/R injury.

AT has been shown to protect many organs, including the heart and kidney, from I/R injury *via* its anti-inflammatory properties (Mizutani et al., 2003; Wang et al., 2013). Unlike traditional regulators of autophagy, such as rapamycin which has many side effects (Sivendran et al., 2014), AT, as an endogenous serine protease inhibitor of the serpin superfamily, may be a safer regulator of autophagy (Ma et al., 2015). The present study demonstrated for the first time that AT attenuates autophagy in a PI3K/Akt-dependent manner in cardiomyocytes.

Taken together, the aforementioned mechanisms expand our knowledge of how anti-thrombin works to lessen I/R injury. Our results showed that AT protected the myocardium from I/R injury by reducing the myocardial infarct size, serum CK-MB level, and expression of apoptotic proteins. Treatment with AT reversed the increased LC3-II and beclin-1 expression *in vivo* and *in vitro*. Interestingly, the expression of p62, a specific autophagic substrate that is degraded during autophagosome processing (Ma et al., 2012), increased after I/R injury in the myocardium but decreased after H/R injury in cardiomyocytes, and these phenomena were reversed by AT. The results of this *in vivo* study are consistent with those of Ma et al. (2012), who showed that p62 expression was increased in myocardial extracts from an I/R (30–90 min) mouse model, indicating impaired autophagic flux. By contrast, the autophagic flux may be “intact” following H/R injury in H9C2 cardiomyocytes. Following I/R injury, myocytes can release tissue factor, leading to the activation of thrombin, which promotes inflammation and apoptosis (Baines, 2011). For this reason, AT may provide anti-inflammatory and anti-apoptotic benefits, which is consistent with our conclusion as well as that of Wang et al. (2013).

Furthermore, our study revealed that AT exerts a protective effect against I/R-induced apoptosis, which involves the AT-mediated inhibition of autophagy. This phenomenon can be explained by the following molecular mechanism. In our study, we observed higher beclin-1 expression, and this effect was reversed by AT treatment. A previous study has shown that ischemia can stimulate autophagy, which is mediated through an AMPK-dependent mechanism. However, I/R can stimulate autophagy, which depends on beclin-1 (Matsui et al., 2007). Moreover, Maiuri et al. (2007) and Oberstein et al. (2007) reported that a pro-apoptotic BH3 domain, sequestered within beclin-1, can bind and inhibit Bcl-XL, indicating that beclin-1 has the ability to induce apoptosis. Beclin-1 may become a pro-apoptotic protein in the case of proteolysis by proteases such as calpain. Moreover, the increased expression of beclin-1 may impede the autophagic flux (Zheng et al., 2017). Other research has shown that impaired autophagosome clearance may contribute to cardiomyocyte death (Ma et al., 2012). Taken together, an inhibition of autophagy induced by I/R injury may reduce cell death and preserve tissue viability.

It has been reported that the PI3K/Akt signaling pathway is activated after myocardial I/R injury, which induces a cardioprotective function (Zhai and Sadoshima, 2012; Yu et al., 2017). This notion is supported by the results of this study, in which the increased ratio of p-Akt/Akt induced by I/R injury was further increased by AT. Surprisingly, the present results indicate that AT downregulates autophagy in I/R injury, in contrast to the results of Ma et al. (2015), who showed that I/R injury markedly increased the expression of p-ULK1, a protein activated downstream of AMP kinase that modulates autophagy (Egan et al., 2011; Laker et al., 2017). The results of Ma et al. reveal the potential of AT to upregulate autophagy, which may be beneficial in the I/R-injured myocardium. However, we showed that autophagy played a detrimental role in I/R injury. A possible explanation for this is as follows. In the study by Ma et al., the LAD was occluded to achieve ischemia for 20 min and then released for 15 min to allow reperfusion. In that study, the duration of surgery was much shorter than that in our study (30 min ischemia and 4 h reperfusion). Different durations of I/R may result in varying degrees of injury (Becker, 2004), and mild injury may induce low levels of autophagy, which may improve cell viability. However, moderate or severe injury may induce medium or high levels of autophagy or autophagic cell death (Matsui et al., 2007).

The main limitation of the present study was the failure to determine the precise mechanisms of how I/R and AT affect the autophagic flux *in vivo*. Therefore, it will be necessary to perform further studies, for example, using chloroquine, a classic autophagy-lysosomal pathway inhibitor (Long et al., 2013), or specific gene knockdown or knockout mice. Although one previous study has shown that the binding of AT to heparan sulfate proteoglycans in the heart is important for its cardioprotective effects (Ma et al., 2015), the identification of the molecule or receptor that AT acts through to regulate autophagy remains to be determined.

This study showed for the first time that AT attenuated myocardial I/R injury by suppressing autophagy in a PI3K/Akt-dependent manner, whereas the activation of autophagy by rapamycin and the inhibition of Akt by A6730 countered these effects. Our data demonstrate AT-mediated cardioprotection against I/R injury and offer a novel viewpoint of the molecular mechanisms of AT.

## Ethics Statement

All animal experiments followed the Wenzhou Medical University Policy on the Care and Use of Laboratory Animals and obtained ethical certification from the Committee on the Ethics of Animal Experiments of Wenzhou Medical University and had therefore been performed in accordance with the ethical standards laid down in the 1964 Declaration of Helsinki and its later amendments.

## Author Contributions

KH drafted the manuscript and prepared the figures. JW and YZ performed the experiments and analyzed the data. SW, LT, YP, and JQ provided suggestions and reviewed the manuscript. YX supervised the study. KJ designed the study, obtained the funding, and supervised the whole project.

### Conflict of Interest Statement

The authors declare that the research was conducted in the absence of any commercial or financial relationships that could be construed as a potential conflict of interest.
